# Some Notes on Phthisis—III

**Published:** 1908-12-19

**Authors:** 


### SOME NOTES ON PHTHISIS—III.

### THE GENERAL APPEARANCE OF THE PATIENT.

The general appearance of patients who come under treatment at a large centre, such as the Henry Phipps Institute,* for phthisis is good in pretty nearly as many cases as it is bad. In a single year it was good in 46 per cent. and bad in 54 per cent. out of a total of 726 patients. Precisely similar figures are afforded by statistics extending over longer periods; thus, in three years, out of 2,210 patients of whom a record upon the subject was kept, the general appearance was good in 47 per cent. and bad in 53 per cent.

Usually a tuberculous subject is supposed to give evidence of his disease in his general appearance, and the consensus of opinion in the past has been that consumption is synonymous with emaciation. This fallacy evidently sprang from the universal failure to recognise tuberculosis in its early stages. Formerly the disease was recognised only when the patient was well upon the path to the grave. Gradually consumption was found to be an advanced stage of what came to be known as tuberculosis; but even then it was usually discovered only when cavities and tubercles co-existed in the same individual. Not until in very recent times has the true beginning of tuberculosis of the lung come to be recognised, and only since then has the general appearance of a tuberculous subject prior to the terminal stage been recorded. As we see the facts now, it would appear that tuberculosis in itself does not always cause emaciation in the individual, and it is only after enough destruction or alteration of tissue has occurred to interfere with the physiological process of the body, or a serious mixed infection has taken place, that marked changes in the individual occur.

As Dr. Flick points out, from what we know now it seems probable that the old ideas of the phthisical habit or the consumptive appearance on which our forefathers laid so much stress was the result of a long poisoning from the tubercle bacillus and from organisms of secondary infection, and not a predisposing condition of the disease as is still widely supposed. The insidious influence of the toxins of the primary and secondary micro-organisms upon the nervous system and upon the nutritive organs of the body, perhaps also upon the development of the muscular system, can apparently bring about the general appearance which has become the classical picture of the inherited predisposition to the disease.

"The bright eye, the delicate form, and the tint of skin which in the past were looked upon as an inherited tendency to tuberculosis we now recognise as the product of the tuberculous toxin."

* *Loc. cit. supra.*

